# Motor Temperature Observer for Four-Mass Thermal Model Based Rolling Mills

**DOI:** 10.3390/s25144458

**Published:** 2025-07-17

**Authors:** Boris M. Loginov, Stanislav S. Voronin, Roman A. Lisovskiy, Vadim R. Khramshin, Liudmila V. Radionova

**Affiliations:** 1 Department of Automation and Control, Moscow Polytechnic University, 38, Bolshaya Semyonovskaya Str., 107023 Moscow, Russia; lb18@yandex.ru (B.M.L.); stsvoronin@gmail.com (S.S.V.); johnkoffee.work@gmail.com (R.A.L.); 2 Power Engineering and Automated Systems Institute, Nosov Magnitogorsk State Technical University, 455000 Magnitogorsk, Russia; hvrmgn@gmail.com

**Keywords:** rolling mill, motor, thermal state, observer, four-mass model, temperature, validation

## Abstract

Thermal control in rolling mills motors is gaining importance as more and more hard-to-deform steel grades are rolled. The capabilities of diagnostics monitoring also expand as digital IIoT-based technologies are adopted. Electrical drives in modern rolling mills are based on synchronous motors with frequency regulation. Such motors are expensive, while their reliability impacts the metallurgical plant output. Hence, developing the on-line temperature monitoring systems for such motors is extremely urgent. This paper presents a solution applying to synchronous motors of the upper and lower rolls in the horizontal roll stand of plate mill 5000. The installed capacity of each motor is 12 MW. According to the digitalization tendency, on-line monitoring systems should be based on digital shadows (coordinate observers) that are similar to digital twins, widely introduced at metallurgical plants. Modern reliability requirements set the continuous temperature monitoring for stator and rotor windings and iron core. This article is the first to describe a method for calculating thermal loads based on the data sets created during rolling. The authors have developed a thermal state observer based on four-mass model of motor heating built using the Simscape Thermal Models library domains that is part of the MATLAB Simulink. Virtual adjustment of the observer and of the thermal model was performed using hardware-in-the-loop (HIL) simulation. The authors have validated the results by comparing the observer’s values with the actual values measured at control points. The discrete masses heating was studied during the rolling cycle. The stator and rotor winding temperature was analysed at different periods. The authors have concluded that the motors of the upper and lower rolls are in a satisfactory condition. The results of the study conducted generally develop the idea of using object-oriented digital shadows for the industrial electrical equipment. The authors have introduced technologies that improve the reliability of the rolling mills electrical drives which accounts for the innovative development in metallurgy. The authors have also provided recommendations on expanded industrial applications of the research results.

## 1. Introduction

Over the past decades, rolling has been developing within the framework of the Industrial Internet of Things (IIoT). Such an approach suggests adopting the processes subject to continuous monitoring, and capable of producing a dynamic reaction to the changes which may influence the production at any state [1,2]. Ref. [3] describes IIoT applications based on the sensors and devices that have been developed for controlling smart industries.

The authors of [4] provide a comprehensive overview of modern AI models used in the IIoT context with a focus on their usage of fault forecasting, process optimization, and predictive maintenance. Some publications suggest using machine learning (ML) methods to evaluate the technical condition of electrical equipment. For instance, the authors of [5] analysed the electrical equipment fault diagnostics system using ML algorithms and conduct simulation experiments to assess the system’s effectiveness. The authors of [6] reviewed feature extraction, classification, training, and testing of models for three different ML methods using an open-source dataset for the subsequent export of results to perform diagnostics on other machines. The authors of [7] developed a data augmentation method that integrates the electromechanical models implemented on a computer. The generalized data on normal and abnormal states are generated using the electromechanical model and a stochastic parameter value selection method.

The authors of [8] state that computer technologies are opening new opportunities for the sustainable engagement of each process equipment piece potential. This confirms that digitalization requires continued monitoring of the equipment technical state, which decreases the industrial accident risks. This point of view is supported in [9], which states that “IIoT provides for the monitoring and forecasting of industrial faults. The idea is to accumulate and visualize data from different industrial parameters by means of introducing IIoT”. The issue is also covered by other papers [10,11,12]. It is stated that one of the top-priority tasks for implementing such ideas is the development of digital models, which mathematically describe the changes of the diagnostic parameters during the facility operation.

New rolling technologies and control algorithms are being developed during the implementation of IIoT applications that provide for the improved product quality [13,14,15]. Digital equipment state monitoring methods are being introduced aiming to extend or preserve the life of the equipment [16,17,18]. Artificial intelligence methods and technical state diagnostic monitoring digital algorithms are applied for the rolling mills electrical drives [19,20,21]. Ref. [22] fairly points that “a rolling mill is an important element in the steel production process, its state directly influences the steel production process”. Thus, studying the rolling mill faults diagnostics is extremely important to improve the production continuity, reliability and safety. Ref. [23] states that “monitoring increases the system safety preventing the risks of ignition, faults and destruction”. This is relevant for the electrical equipment and roll mill stands motors. Some papers on the issue dwell upon the development of the systems for the state monitoring and identification of the synchronous machines faults [24,25,26]. However, operating high-capacity motor thermal state continuous control has not been developed yet. The research is mainly performed in laboratories and does not result in industrial applications.

The development of smart systems for on-line state monitoring implies the use of digital shadows that serve as coordinate observers for the electromechanical and mechatronic systems. The authors of [27] reference [28] and provide the following definition: “A digital shadow is a unilateral automated data stream from a real object to a digital one that is usually determined as an emulation of a physical asset or process. The typical tasks associated with it include real-time modeling and process monitoring”. Ref. [29] describes the concept of object-oriented coordinate observers that characterize the technical state of an industrial facility. They are developed using software that does require specialized digital platforms. The application of digital shadows for the thermal state control of rolling mill motors being the most responsible equipment has been substantiated. It is expedient to accept this approach for the development of the on-line temperature monitoring system for the motors of plate mill 5000.

### 1.1. Substantiation of the Study

**Figure 1:**
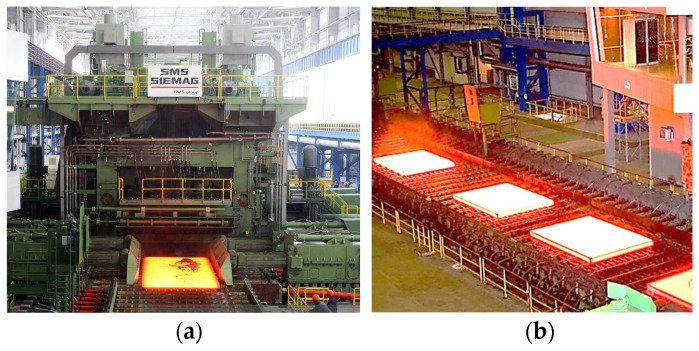
Workpiece rolled by roll stand of plate mill 5000 during roughing rolling (**a**) and cooling-down of workpieces at roller table (**b**).

Rolling mill motors are generally unique (custom-designed) and powerful; they are quite frequently low-speed and water-cooled. Usually, they are among the priciest assets of a metallurgical plant. This is applicable to the electrical drives of stands in plate mills 5000, as well. Such drives are used in many countries. Rolling on them is performed in the reversible mode in the horizontal and vertical stands that constitute a single complex. The photo of a horizontal stand is given in Figure 1a. For the thermomechanical rolling technology, the process is divided into two stages: roughing that includes 5–6 passes and finished product rolling that includes up to 19 passes. During roughing, several workpieces are rolled with intermediate cooling-down at the roller table (Figure 1b) in accordance with the thermomechanical rolling [30,31]. During finished product rolling, each sheet is rolled to its final thickness. Here, workpiece means a semi-finished product between the initial slab and the final product.

Individual electrical drives based on synchronous motors with frequency regulation are installed at the horizontal stand of plate mill 5000. This allows for the independent control on the upper and lower rolls that is required for the formation of a ski bend at the front part of the workpiece [32,33]. However, the ski bend formation is challenging for levelling (adjustment of) loads at the motors of these rolls in the basic rolling mode (see the proof below in Section 2.1). Mostly, adjusting motor loads is impossible. When short workpieces are rolled during the roughing, loads are not levelled at all (the reasons are considered in [34]). This results in the violation of thermal regimes and uneven motor heating.

Some of the problems have been already studied in the research that aimed at the control of the motor loading modes. The authors developed a method for calculating equivalent torques (and currents) immediately during the rolling process [29], and systems for controlling thermal regimes have been introduced [35]. The development of observers for calculating (recovery) the temperature by means of changing stator and rotor current has been scientifically substantiated. The temperature observer for stator windings and iron core based on a two-mass heating model has been developed, while practice has shown that the capabilities of such an observer are restricted. It does not provide enough information to control the motor thermal state. This is explained by the fact that hot rolling mill stands motors generally operate in tough environments, often at ultimate loads. Thus, up to 240% overload is permitted for the electrical drives of a rolling mill 5000 stand. That is why the torque limit is frequently hit during passes. In addition, the range of product sizes of such rolling mills is extending to the rolling sheets of hard-to-deform steel grades. Moreover, cobbing and rolling speed may vary.

In many cases powerful rolling motors are equipped with air-to-water cooling system, and two-mass temperature control, offered in [29], is not enough. Thus, the objective is to develop a thermal state monitoring system based on measurement of a larger number of parameters.

The main requirements for the developed system are as follows:

- simple operation, because it will be used in industrial conditions rather than in-lab;
- no complex computational algorithms and mathematical methods;
- easy in operation and user-friendly, and can be developed based on generally available software;
- ensuring temperature control for main nodes (components) of the motor on-line.

Taking these requirements into account, it is preferable to use the models from the MATLAB Simulink package. Apart from the specified temperatures measured in the two-mass models, it is necessary to control rotor winding and iron core heating. Quite frequently, the applied bearing thermal state control [36,37,38] is not critical, because the rotation frequency of plate mill motors commonly does not exceed 70–120 rpm. Casing and flanges temperature control is not informative because of their large size and satisfactory external cooling of the motor.

The article solves the task of building a system matching the listed requirements.

### 1.2. Relevance of Motor Thermal Regimes Control

The off-limit temperature increases that result in the deterioration of insulation are a frequent reason of faults in electrical machines. The stator and rotor windings, the stator magnetic circuit overheating can deteriorate the motor operational parameters [39]. This reduces its life unless the load is duly distributed. This conclusion is important for motors of the upper and lower rolls (UMD and LMD) main drives in plate mill stands. In Section 2.1, it is shown that the motors of the rolling mill 5000 stand in the majority of passes work with different loads, varying three-fold or more. The load ratio during rolling changes. This overloads and overheats one of the motors. When limit temperatures are exceeded because of high heat loss, insulation materials of the winding are worn out excessively and irreversibly. This has a negative impact on the electrical strength of the insulation and its ability to withstand short circuits. Eventually, this shortens the motor life and results in its breakdown. Thus, temperature is an important factor influencing the lifetime of motors.

The following theories that are true for alternating current were confirmed by the research [40]:

1. High temperature results in increased copper resistance, and consequently, causes increased losses in windings.
2. Inefficient cooling system results in quick insulation deterioration and more frequent failures of windings because of the increased heat loss.
3. Thermally non-conductive materials, such as winding insulation, can produce local heat concentration.

The level of limit temperature depends on the thermal resistance class of the winding insulation and can vary from 90 °C to 180 °C. Ref. [41] establishes limit values for the exceeding temperature of the active parts of powerful electrical motors at ambient temperature of 40 °C (Table 1). Similar limit values for stator windings depending on insulation classes were set by the National Electrical Manufacturers Association (NEMA) [42,43]. Strict norms must prevent excessive thermal stress and ensure a long-term and reliable operation of the motor.

The authors of [39,40] state that, when the operating temperature of an electrical machine increases by 10 °C, its lifetime decreases by a factor of two. A similar conclusion was drawn in [44]: “insulation heating by every 8 °C in excess of the limit value reduces its life twice. That is why any increase in current loads by more than 10% must be avoided”. And that “the most effective heat management increases the productivity and provides for the reliable operation of the electrical machine, but poor heat management, on the contrary, can cause machine failure” [40]. Such a requirement cannot be set for the electrical drives of the rolling mill 5000, because the range of its operational loads exceeds the nominal torque and, consequently, the current, twice over. This additionally confirms the need for the on-line motor temperatures monitoring.

Motor temperatures are influenced by the thermal state of each part and the environment [45]. The authors [46] stated that “the temperature of stators and rotors in large-size machines is important for the short-term protection and for the long-term monitoring of their state”. Apart from winding temperature rise, iron loss is another common reason for the electrical machines overheating. As a rule, they are predominant when the machine operates at high frequency. According to ref. [47], limit temperature values are set for the basic elements, winding, and iron core of high-capacity electrical machines accounting for their insulation class (Table 2).

The lack of natural cooling requires additional control of thermal regimes. Failures or temporary faults of air-to-water cooling result in overheating and reduction of the motor reliability. If the temperature of the main elements is not monitored, it often becomes difficult to determine the reason of overheating, unless it has already caused obvious destruction of insulation or damaged the electrotechnical steel. That is why it is important to control stator and rotor heating on-line along with the winding temperatures. Thus, on-line thermal state monitoring systems should use multiple-mass thermal models. This requirement was taken into account for the development of the motor temperature observer considered in this article. As in [29], the studies are applied to the motors of the horizontal stand of the rolling mill 5000. Finally, there are recommendations on the introduction of the developed technologies for other machines. Hence, a short overview of publications in the considered field of research is provided below.

## 2. Literature Review

The authors of [48] review and consider the issues of monitoring and maintenance of equipment in production lines. With reference to [49,50], they state that well-maintained equipment is the basis for continuous production. To avoid failures, it is necessary to control the equipment state immediately during operation [51]. It is also mentioned that “conventionally, the facilities are controlled manually and maintained after faults” [52,53]. However, such a strategy cannot exclude the negative impact of the equipment downtime on the productivity and implies high costs [54]. Thus, monitoring the in-operation state of the electrical machines is especially urgent. Previously, it was solved by installing special sensors designed to measure the winding temperatures.

### 2.1. Systems Based on Temperature Sensors

Special temperature sensors were developed to control the thermal state of motors. The most difficult part is to install temperature sensors on rotating elements. Thus, ref. [55] offers a device measuring the local surface and inside rotor temperatures that gives a relatively precise three-dimensional thermal pattern of the rotor. The applied structure includes up to 12 simultaneous local temperature measurements at random places of the rotor. However, due to the problems with the assembly that are described in that article, “the applicability of the system is obviously restricted by the laboratory testing environment”. Thus, the device is not suitable for use in industrial motors. Refs. [56,57,58] consider sensors and temperature measurement systems for synchronous motor rotors with permanent magnets (PM) that are not applicable in powerful electrical drives of rolling mills either. Ref. [59] states that “equipping the rotating element with thermal sensors is expensive and is not representative for large-scale manufacturing”.

Based on the research described in [60], it was concluded that “the quantity of temperature sensors and their location are often restricted because of the physical boundaries and the equipment cost, and the temperature of the majority of the motor parts cannot be measured directly”. Ref. [61] states that “it is better to avoid temperature measurement using sensors both in the stator and in the rotor at all or if possible”. Thus, the industry and the research institutes require temperature measurement methods based on mathematical models integral to the observers. Methods used to determine the parameters of thermal models are covered in [62,63].

### 2.2. Analysis of Known Temperature Observers

One of the relatively early publications on the issue [64] describes a microprocessor device for thermal forecasting used in asynchronous motors providing the protection from and management of motor ageing. They use a linear model with lumped parameters to describe the dynamic thermal characteristics. The model was developed based on the information on the dimensions and constant thermal coefficients, so it can be applied to any similar machine. Thermal calculation is performed in real time by a microprocessor that evaluates the motor temperature based on the primary inputs of phase current and ambient temperature. The results were demonstrated for a 5.5 kW machine. Ref. [65] shows an observer for temperature evaluation in electrical machines based on the thermal model implemented in a microcomputer. It required only the measurements of current, voltage, speed, and temperature, available from an easily accessible place. The article shows the results of several experiments including one for the motor of a 3500 kW rolling mill.

The authors of [66] describe a temperature evaluation algorithm (thermal observer) that evaluates the thermal state of an electrical machine in real time. It is a Kalman filter that unites the forecast of the electrical machine heating based on the thermal model with lumped parameters. It uses temperature measurements with one external sensor. Ref. [67] develops this idea further, offering a virtual thermal sounding. They developed a virtual thermal sensor for evaluating temperature in different parts of the electrical machine. It is a dynamic observer based on the model that required only one temperature sensor installed in an easily accessible place. The authors of [68] presents an adaptable on-line forecaster of stator winding temperature applicable in asynchronous machines. They described a hybrid thermal model that accounts for the differences in thermal operation conditions of different motors having the same power and the same design equipped with fan cooling. The experiments confirmed the efficiency of temperature evaluation based on the on-line parameter adjustment.

A number of developments were implementing the MATLAB Simulink resources. Thus, ref. [39] considers a thermal model used to assess the stator and rotor temperature in an asynchronous motor with a squirrel-cage rotor. The thermal model accounts for the dissipated power, heat transfer, and temperature increase speed in the stator and rotor. Non-linear electrical behaviour and thermal state equations are solved using the MATLAB Simulink blocks. This allows determining the temperature in stator and rotor windings and checking the state of insulation in different operating conditions. The analysis results suggested that the winding temperature increases because of the losses in the stator and rotor copper. Stator current increases because the electrical drive system reacts to the increased torque depending on the load, and the temperatures of each element increase as well.

The authors of [69] describe the implementation of the mathematical model of interrelated electromechanical and thermal processes in a linear asynchronous motor using MATLAB Simulink. The developed model consists of an electromechanical model, a thermal model, and a model of hydraulic cooling system that influence each other. The calculation results utilizing the model, developed on the detailed thermal equivalent circuit, demonstrate close agreement with the finite element model. They determined the safe operation range for the asynchronous traction motor accounting for uneven longitudinal heating with different cooling systems and heating medium consumption.

Both developments lack experimental studies. But, they demonstrate that it is expedient to use MATLAB Simulink for the evaluation of thermal state of different motors.

In addition, a considerable part of the studies is devoted to the development of thermal models for synchronous machines with permanent magnet motors. They are widely used in the electrical vehicles popular today. The analysis and maintenance of thermal regimes of permanent magnet motors are widely covered by publications. This is confirmed by [70], which gives an overview clarifying and assessing the existing thermal monitoring methods. High temperatures are the reason for many typical damages and failures, such as permanent magnet demagnetizing and turn-to-turn short circuit in stator winding. Thus, temperature control for these elements is required to provide a reliable motor operation. The issues are covered in [71,72,73] and other publications.

### 2.3. Conclusions to the Review

1. The general disadvantages of the known developments are as follows:
  - Their implementation in the operating equipment installed at production lines is complicated;
  - They lack experimental proofs and practical industrial evaluation.

The developed algorithms for heating control have been studied either by mathematical models or in laboratories. The publications provide no information on their practical implementation.

2. As of today, there are almost no developments of observers designed to control the temperature of powerful rolling mills synchronous motors. The majority of studies are performed for PM synchronous motors installed in vehicles. Quite frequently, they solve the problem of preventing the permanent magnet demagnetizing with the view of avoiding the motor overheating [74]. Or, on the contrary, the problem is to analyse the temperature impact on the permanent magnet properties. Ref. [75] states that “such factors as insufficient dissipation of heat and extremely high temperature can easily result in the PM demagnetizing”. For synchronous motors with electromagnetic excitation these problems do not exist.
3. A promising development is to make observers based on digital models. Such an approach opens up opportunities for the development of temperature observers based on domains that are included into widespread thermal process simulation software packages. The advantage of such approach is that it does not require complex mathematical tools. The second advantage is that it determines a minimum quantity of model parameters by well-known methods that is important for the electrical drives that are currently in operation.
4. Taking into account the development and availability of software, it is expedient to use multi-mass models based on the MATLAB Simulink resources. The performed analysis demonstrated that the development of a plate mill motor temperature observer requires the development a four-mass model, which would suffice. Below we will show that the development should be performed on the basis of the Simscape Thermal Models library domains from the Simulink package.

## 3. Problem Statement

### 3.1. Studied Object Description

**Figure 2:**
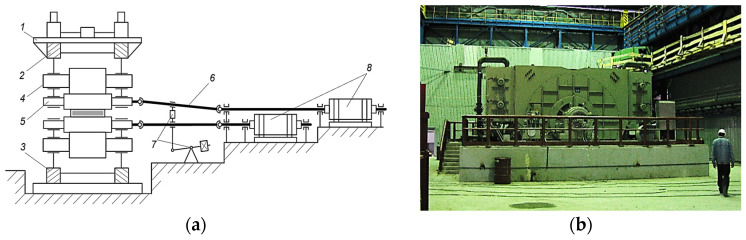
Custom electrical drive of horizontal stand (**a**) and photograph of mill 5000 motor (**b**): 1 is the frame; 2 and 3 are electromechanical and hydraulic screw-downs, respectively; 4 and 5 are backup rolls and work rolls, respectively; 6 are retractable spindles; 7 is counterbalance; 8 are motors.

As it was mentioned earlier, UMD and LMD of horizontal stands in plate mills are designed individually without speed reducers. A simplified kinematics scheme for the rolling mill 5000 stand is given in Figure 2a. During rolling, the rolls are rigidly connected through metal, and any speed mismatching results in the redistribution of loads (torques and currents) of the motors. This leads to the differences in root–mean–square currents and, consequently, to the differences in the heating of the motors.

The upper roll motor photo is given in Figure 2b. The synchronous motor VEM DMMYZ 3867-20V was installed. Its characteristics are given in Table 3. Frequency converter characteristics are given in [76].

According to the table, the motor is low-speed; nominal rotation frequency is 70 rpm. A more than two-fold overload in torque and current is allowed which sets strict requirements for the control of thermal regimes and operation of the cooling system. IC86W stated in the last line of the table means a fully enclosed motor with independent air/water cooler. A cooling system with forced ventilation inside the stator and a water-cooled heat exchanger is applied. The system characteristics are given in [77]. The review of the most widespread cooling systems for motors is given in [78].

**Figure 3:**
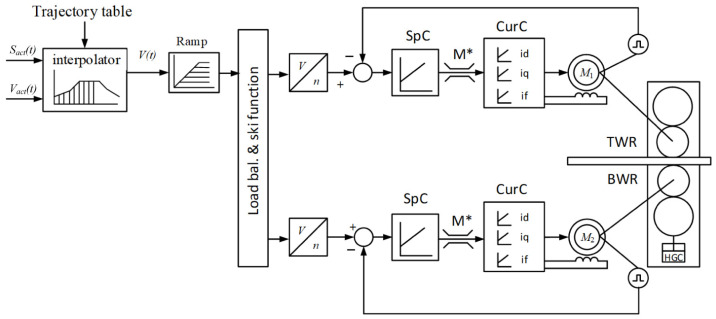
Structural scheme of speed control for horizontal stand electrical motors.

UMD and LMD speed setting scheme is given in Figure 3. The structure has the Load bal. & ski function block that splits loads and ski formation. It is responsible for the formation of the ski bend at the front part of the workpiece when it leaves the stand. This mode is envisaged for the majority of hot rolling stands [32,33]. The terms “ski” and “ski effect” are common in the theory of rolling and rolling mill production. It is used in study guides and research publications on metal sheet rolling, particularly in articles [29,79,80]. They feature photographs of bent front edges of workpieces produced by the 5000 mill that look similar to a ski. After the implementation of the function motor loads are split (adjusted) [81,82]. However, in practice, the project electrical drive control algorithm is not quick enough in this mode [83]. Ref. [29] shows that the mismatching of speeds is not adjusted in most of the passes. This results in a many-fold difference of torques and currents in motors with consequent adverse effects.

**Figure 4:**
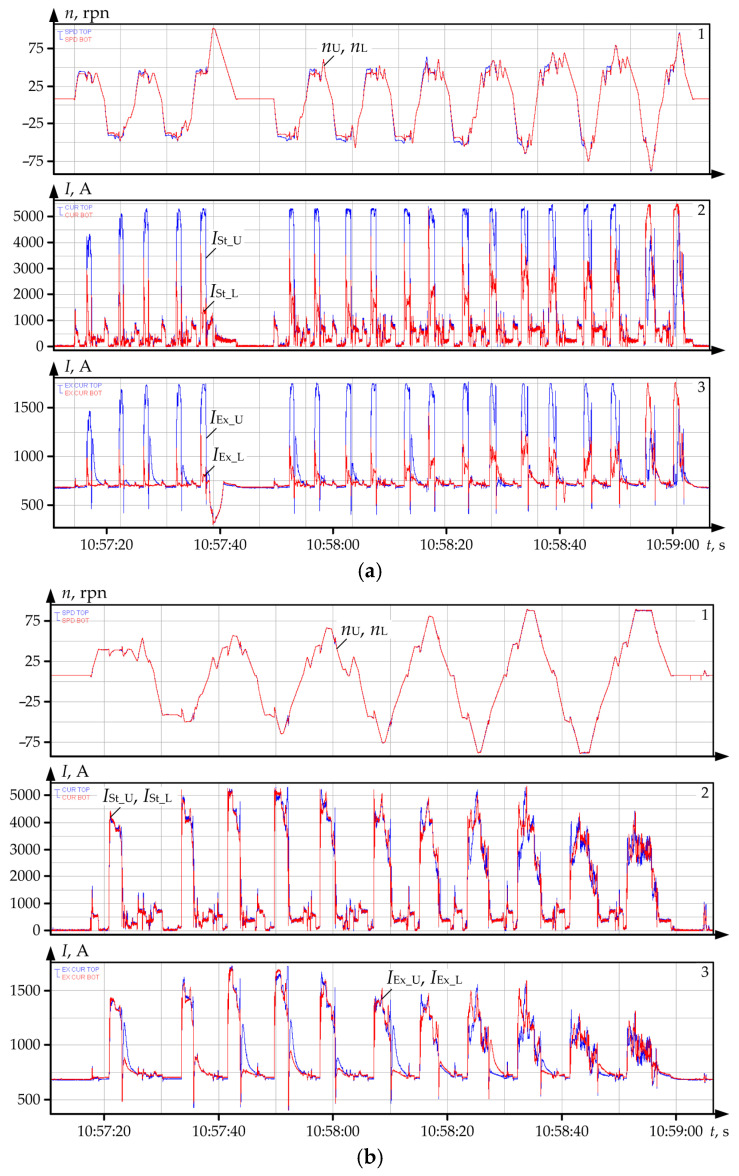
Oscillograms of speeds and currents of UMD motors (blue) and LMD motors (red) during rolling with a 10% mismatching of speeds (**a**) and with equal set speeds (**b**); panel 1—speeds *n*_U_, *n*_L_, rpm; panel 2—stator currents *I*_St_U_, *I*_St_L_, A; panel 3—excitation currents *I*_Ex_U_, *I*_Ex_L_, A.

It is confirmed by the fact UMD and LMD motors that in the majority of the passes operate at different loads and demonstrate oscillograms shown in Figure 4. The oscillograms in Figure 4a were received during the rolling of workpieces with a large initial mismatching of speeds in each pass (with a 10% ski setting). Similar oscillograms in Figure 4b were received during tolling with equal initial speeds, i.e., without ski setting. Panel 1 shows the oscillograms of the set and actual speeds of UMD and LMD motors. These dependences coincide, that is why they have not been marked. Panel 2 shows currents of stators *I*_St_U_, *I*_St_L_, while panel 3 shows oscillograms of excitation currents (rotor currents) *I*_Ex_U_, *I*_Ex_L_ of motors.

Oscillograms in Figure 4a confirm the conclusion on many-fold difference of the stator currents and, consequently, of UMD and LMD torques. Current *I*_St_U_ of the upper roll motor exceeds current *I*_St_L_. In the last passes, the correlation between the motor roads changes (*I_St_L_* becomes higher than *I*_St_U_). This is explained by the operation of the load splitting function, because with higher length of the workpiece ski formation system switches off, and the splitting function starts operation [29]. The validity of the conclusion on the influence of the electrical drive speeds mismatching on currents and torques is confirmed by oscillograms in Figure 4b. They were received with equal set motor speeds, i.e., for rolling without ski formation. Thus, their speeds during all passes are equal. Consequently, stator currents (panel 2) and excitation currents (panel 3) coincide, as well.

The following conclusions were drawn following the comparison of these figures:

1. During rolling with the set ski (Figure 4a), the established stator current *I*_St_U_ of UMD motor (panel 2, blue) in the majority of passes reaches the limit by current equalling 5200 A. It is more than two times higher than the nominal motor current equalling 2460 A (ref. to Table 3). The equal current *I_St__*_L_ of LMD motor (panel 2, red) in 17 passes does not exceed half of the nominal value. The correlation of currents changes only in the last two passes. The reasons for this fact are related to the changes in the thickness of the rolled workpiece, they are considered in [84,85,86].
2. The same conclusion can be drawn from the analysis of excitation currents (panel 3). Quasi-steady values of excitation currents *I*_Ex_U_ of UMD motor in the major part of the passes are approximately 1700 A, and they exceed the maximum value of 1680 A (Table 3). At the same time, similar values *I*_Ex_U_ of LMD motor are approximately 700 A, which is lower than nominal current equalling 898 A.
3. During rolling with equal set speeds (Figure 4b) the average currents of stator (panel 2) and rotor (panel 3) for UMD and LMD are equal and do not exceed the limit values. However, it should be noted that the regime of total coincidence of speeds in all passes is ideal and cannot be implemented in practice. Consequently, currents of UMD and LMD motors almost always differ. As it was said, this is explained by the formation of ski.
4. Excitation currents make approximately 30% of stator currents, thus, losses for excitation are significant. Consequently, heating of the rotor winding and of the rotor iron core should be taken into account for the analysis of the motor thermal state.

The described analysis suggests that due to the difference of the UMD and LMD motor currents their thermal regimes will differ, as well. The stator winding and the rotor winding will have different temperatures. The stator iron core and the rotor iron core will heat up differently. This makes us draw an important conclusion: apart from the temperature control of stator winding and iron core suggested in [29], it is necessary to constantly monitor the same temperatures for the rotor. A significant change in these coordinates is conformed below in Section 6.

### 3.2. Known Thermal Model Based on Analytical Formulae

This situation brings about the development of an object-oriented coordinate observer for motor temperatures in rolling mill 5000. Ref. [29] gives its description, and the effectiveness of on-line stator temperature monitoring is confirmed there. However, this development has a significant disadvantage: it is based on the two-mass thermal mode consisting of heat absorption of the stator winding and iron core. Thermal flow for each thermal mass:

(1) $$Q=Cm\frac{dT}{dt},$$

where *Q* is heat flow in W; *C* is heat absorption; *m* is weight in kg; and *T* is temperature of the mass.

Heat exchange between masses is determined from thermal conductivity between winding and iron core through insulation and convection between rotor iron core and stator iron core. Heat exchange from thermal conductivity is described by equation

(2) $$Q=k_{1}\frac{A}{D}(T_{A}-T_{B}),$$

where $k_{1}$ is thermal conductivity of the material between masses (in our case, insulation), W/(m °K); *A* is the area through which heat is transferred; *D* is the thickness of the layer through which heat is transferred; $T_{A}$ is the temperature of layer A (mass 1), °K; and $T_{B}$ is the temperature of layer B (mass 2), °K.

In general case, heat exchange by convection is described by the following equation

(3) $$Q=k_{2}A(T_{A}-T_{B}),$$

where $k_{2}$ is heat transfer by convection coefficient, W/(m °K); $T_{A}$ is temperature of body 1, °K; and $T_{B}$ is temperature of body 2, °K.

**Figure 5:**
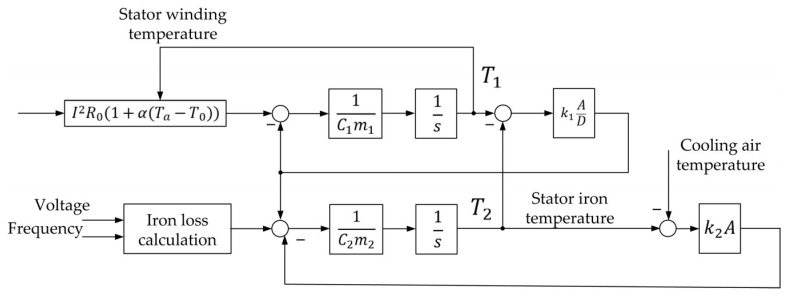
Structural scheme of two-mass thermal model of motor.

The structural scheme of two-mass thermal mode developed on the basis of these equations is given in Figure 5 [29]. Its use can be recommended when the resource of the programmable logic controller (PLC) of the stand is limited, thus the application of a complex heating model is complicated. At the same time, it was shown above that a more precise four-mass thermal model is required for full-scale monitoring of a powerful synchronous motor.

### 3.3. Research Objectives

Based on the above the authors have determined the purpose of the study: to develop and adopt a thermal state observer based on four-mass thermal model for synchronous motors of plate mill stands.

The following objectives have been set:

1. Develop the motor thermal model that accounts for the heating of stator and rotor windings and iron core.
2. Perform virtual setting of the observer in rolling mill 5000 electrical drives.
3. Check the model adequacy by comparing the motor temperature with the actual values measured physically with a sensor. But it is not possible to receive such information from the motors of the main electrical drive of the rolling mill 5000 horizontal stand (and from the majority of other industrial electrical drives) because they do not have stationary sensors. That is why it is better to measure the temperature in control points at windings and iron core of stator and rotor using a portable device (pyrometer). Further, it is required to compare the results with the temperature from the oscillograms received from the developed thermal model.
4. Study the thermal state of specific masses at different loading of UMD and LMD motors. Analyse the motor temperature during rolling of several batches of sheets.

## 4. Materials and Methods

During the development of the motor thermal state observer the authors have applied the method of virtual launch described in [87]. The method implies the simulation of processes in the model with further virtual adjustment at the facility using hardware-in-the-loop (HIL) simulation. The method includes the development of the simulation model in the MATLAB Simulink or Simscape software, followed by loading to the PLC software. The information is exchanged with the facility by means of exporting data from the signal archiving system that is part of the process data acquisition system (IbaPDA) installed at the mill [88]. Further on, at the stage of the virtual observer commissioning, the PLC is connected to the facility physically. Computational algorithms are corrected, and the setting parameters are adjusted.

Below is the method for determining the stator and rotor windings and iron cores temperatures for the studied salient-pole synchronous motors with water cooling. It is applicable both to on-line temperature recovery and to the processing of earlier prepared sets of electrical parameters of motor that influence its temperature.

The method includes the following:

1. Reading the data that characterise the thermal motor state obtained by direct measurements and saved to the IbaPDA system archive. When necessary, smoothing and averaging of data using statistical processing algorithms.
2. Data export from the system to the MATLAB software file. Adjustment and testing of temperature recovery algorithms for specific masses from the four-mass model using the HIL simulation.
3. Recovery of thermal parameters for the set time period. Motor heating is a long-term process, so we recommend the following periods: all roughing or finished product rolling, full cycle, several cycles.
4. Validation of the results by comparing with the data obtained by direct measurement of the temperatures of specific masses immediately at the motor. Results averaging and comparing with limit values in accordance with the winding insulation class.

**Figure 6:**
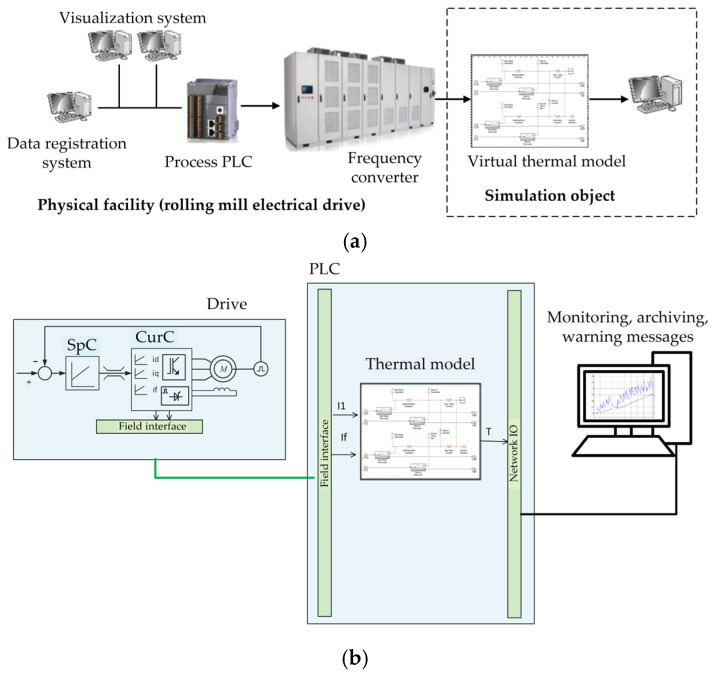
Structure explaining HIL simulation for setting of observer (**a**) and structure of motor thermal state observer (**b**).

HIL simulation was applied for the model testing and validation [89]. It is described by Figure 6a, which shows the location of the simulation object at a separate computer. It is convenient for setting the computational algorithm when using complex virtual models that provide for a high precision of the process simulation. The structure of the motor thermal state observer is given in Figure 6b. It was developed on the basis of the four-mass heating model described in the next section. The measured (or earlier recorded) coordinates of the electrical drive that determine the thermal state of the facility are supplied with a fixed increment to the inputs of the virtual model in the MATLAB package. These coordinates are the stator and rotor currents.

Further on the adjusted thermal mode is loaded to the PLC structure of the stand. The data are exported from the IbaPDA system to the file that is imported into MATLAB. After that the developed observer is used to control the motor thermal state right during the rolling process. The described approach was used to virtually commission several metallurgical plants. In addition, it was applied to adjust the elastic torque observers at drive shafts [90,91] and the angular play observer in spindle connections of the mill 5000 stand [92]. The advantage of such approach is its high precision with minimum commission time.

The structures shown in Figure 6 schematically show the universal thermal model developed on the basis of the Simscape Thermal Models domains. Similar thermal models are considered in [93] where basic thermal blocks and simulation models are described. Domain facilities are classified by categories:

- Thermal elements are thermal building blocks such as thermal mass, different blocks of heat transfer;
- Thermal sensors include temperature and heat flow rate sensor blocks;
- Thermal sources are temperature and heat flow rate source blocks;
- Thermal systems are modular structures that are examples of thermal systems.

Thermal libraries contain blocks for a thermal domain organized as elements, sources, and sensors. The combination of such blocks allows receiving the required thermal model and studying physical processes. It is also recommended to use these blocks together with the blocks from other Foundation libraries for modelling multi-domain physical systems.

Multi-mass thermal models are built on the basis of the mentioned blocks using computing functions. The Simscape Thermal Models documentation mentions seven- and nine-mass models. Ref. [94] considers a seven-mass thermal circuit of the motor with lumped parameters, describing the model, its structure, and the results of the simulation. It implemented the modules of rotor and stator winding and iron core, bearings, flange, and casing of the machine. For that model, the motor thermal circuit consisted of thermal conductivity, thermal masses, and blocks of thermal exchange by convection that recreate thermal paths in the motor parts. The motor exchanges heat with the atmosphere via contacts casing–atmosphere, flange–atmosphere, and back plate–atmosphere. The same publication mentioned that, if necessary, it is possible to switch to a nine-mass model, including the motor shaft and the frame separately.

However, the use of the seven- and nine-mass models is complicated by the necessity to determine its parameters: masses of the listed elements, heat absorption of each mass and other coordinates included to the model. In addition, the information given by such models is excessive for operating motors. Calculation of the flange temperature, bearing plates, etc. is important for the development and designing of electrical machines, but for assessment of the motor current state in operation it is not required. Also, validation of complex thermal models is difficult, because it required temperature control in the point that are not easy for access. Accounting for the described disadvantages, it is expedient to develop a four-mass model that was accepted for the implementation and is characterized below.

## 5. Implementation

The development of a thermal model (virtual temperature sensor) based on the Simscape Thermal Models domains has the following advantages:

- The developed model is simple and lean;
- It can be conveniently tied to the developed rolling process models and to the actual values of the coordinates measured at the real facility (currents, voltages, speeds).

**Figure 7:**
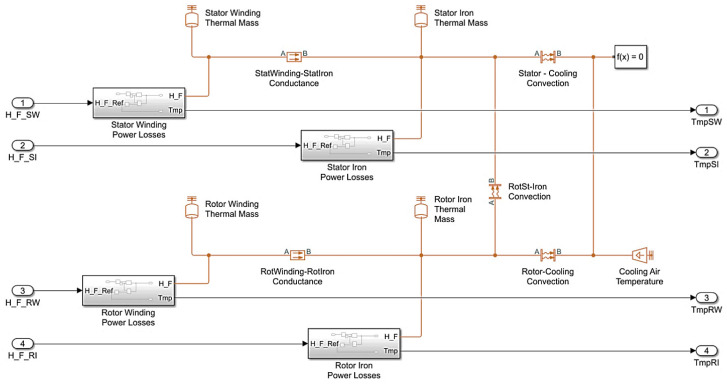
Scheme of four-mass thermal model for mill 5000 motor.

The model structure is given in Figure 7. The information on specific thermal masses used there is given in Table 4.

Figure 5 and Figure 7 suggest that a two-mass thermal model includes the heat capacities of the winding and the stator hardware, while a four-mass model also considers the heating of the winding and the rotor hardware.

The expressions in the table were taken from the description of Simscape Thermal Models domains [93,94]. The emissivity equation is not used in this library, and therefore, it is not reviewed in the model. Coefficients k_1_ and k_2_ were taken from literature, no experimental calibration was performed. Following the recommendations from [93], the thermal conduction of the material between the masses was set (by default) at k_1_ = 401 W/(m∙°K); and the convection heat transfer coefficient k_2_ was set at 20 W/(K∙m^2^) [95].

Note that the coefficients $k_{2}$ and *h* use the same definitions and measurement units, which is correct. The difference between them can be explained by Newton’s cooling law. The authors of [96] provide the following explanation: “The relevant boundary layer heat transfer mechanism is viewed as conductivity through stationary liquid (or gas) near the wall, which equals the convection speed from the boundary layer of the liquid or gas”. This can be written down as follows:

(4) $$hA(T_{S}-T_{f})={-k}_{2}A(dT/dy)S$$

where *T_S_* is the temperature of the solid surface (hotter medium); *T_f_* is the free stream temperature of liquid or gas (cooler medium); and *y* is the coordinate perpendicular to the emitting surface plane.

Thus, according to (4), the convection coefficient can be assessed by measuring the rate of heat transfer (in this case, it is the coefficient $k_{2}$) or difference of temperatures (coefficient *h*). In the literature, these coefficients feature the same measurement units, although coefficient *h* is not a thermodynamic property. It is a simplified proportion for the state of gas or liquid and is therefore often referred to as the stream characteristic.

**Figure 8:**
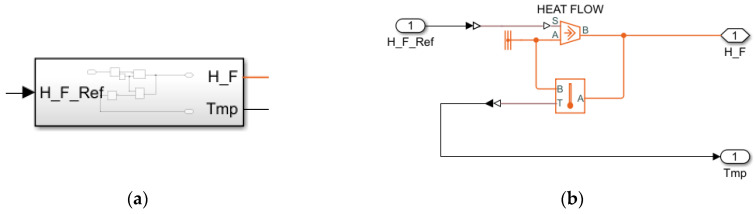
Block (**a**) and structure (**b**) of heat flow transfer model.

The model contains four masses: stator and rotor windings, stator and rotor iron core represented as the following sub-systems:

1. Heat flow transfer system (heat emission in windings and iron core) (Figure 8a). Each block is a structure shown in Figure 8b.
2. Heat emission in the winding during current flow is included by calculation as per the equation:
  (5) $$P=I^{2}R_{0}\left(1+\alpha \left(T_{a}-T_{0}\right)\right),$$
  where P is the heat emitted in the winding; $R_{0}$ is resistance at the ambient temperature $T_{0}$; $T_{a}$ is actual winding temperature; α is temperature coefficient of winding material resistivity, W/(m °K); and I is current flowing through the winding.
3. Heat emission in the stator iron core. Losses in the synchronous motor stator steel at variable frequency and flow are determined by the formula [97]:
  (6) $${∆P}_{st1}={∆P}_{st1Nom}{\left(\frac{F}{F_{Nom}}\right)}^{2}{\left(\frac{f_{1}}{f_{1Nom}}\right)}^{m}$$
  where ${∆P}_{st1Nom}$ is nominal value of losses in the stator steel; $F_{Nom}$ and F are nominal and actual flows, respectively; $f_{1Nom}$ is nominal frequency of the stator current; $f_{1}$ is actual frequency of the stator current; and m is the exponent than can have the value 1.3–1.5 depending on the steel grade.

**Figure 9:**
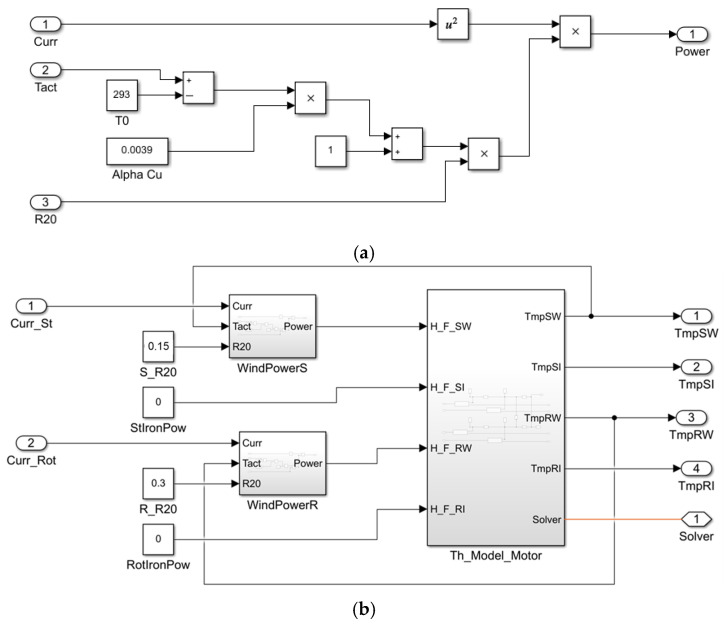
Structures of winding heating model (**a**) and of motor four-mass thermal model (**b**).

Equation (5) is implemented in the model (Figure 9a). The general structure of the motor thermal model is given in Figure 9b, and its parameters are given in Table 5.

**Figure 10:**
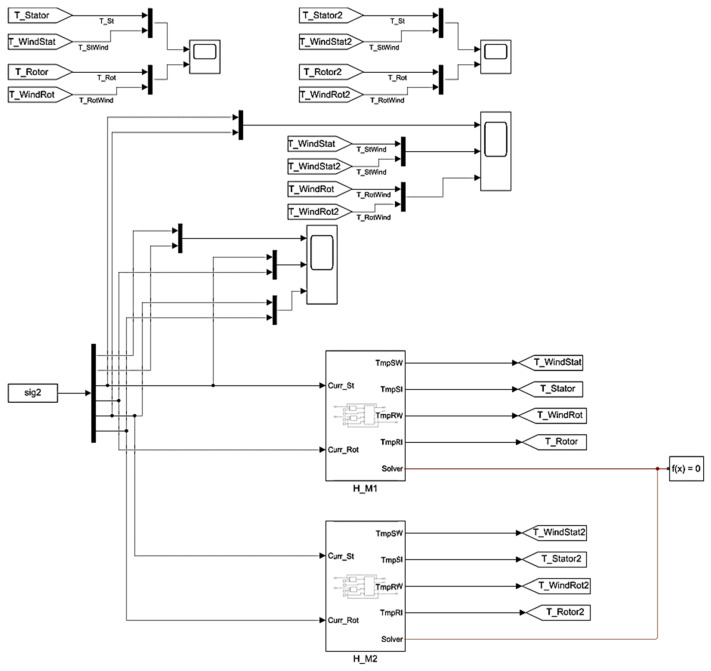
Structure of thermal model of two rolling mill stand motors in MATLAB Simulink.

Using the described structures and thermal parameters, the authors have developed a heating model for two motors in the Simulink software package that is shown in Figure 10. Here, the thermal models are represented by blocks H_M1 and H_M2, and other elements are generally accepted for the MATLAB Simulink software.

According to the set objective, at the next stage, the authors analysed the heating of separate masses and checked the adequacy of the results of their temperature restoration.

## 6. Results

### 6.1. Results Validation

**Figure 11:**
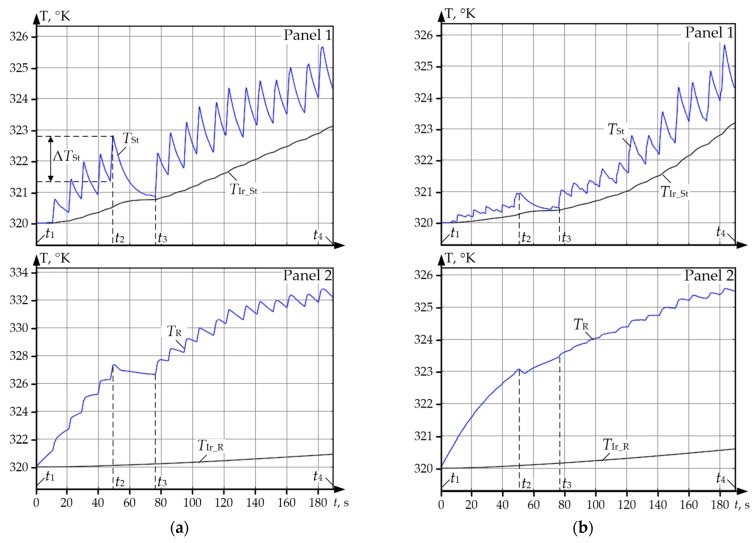
Diagrams of temperature of the UMD (**a**) and LMD (**b**) motor masses restored by observer: panel 1—stator winding temperature (*T*_St_) and stator iron core temperature (*T*_Ir_St_); panel 2—rotor winding temperature (*T*_R_) and rotor iron core temperature (*T*_Ir_R_), (°K).

Using the developed observer, the authors analysed the thermal modes of motors against the datasets corresponding to the oscillograms given in Figure 4a. The results are given in Figure 11a,b for the upper and lower roll motors, respectively. Temperature diagrams for separate masses of the four-mass system were built from them. In Section 2.1, it was stated that they correspond to rolling with a large pre-set ski, that is why average loads in all passes differ. Consequently, the motor temperatures in the analysed (and a relatively short) time period are different.

**Figure 12:**
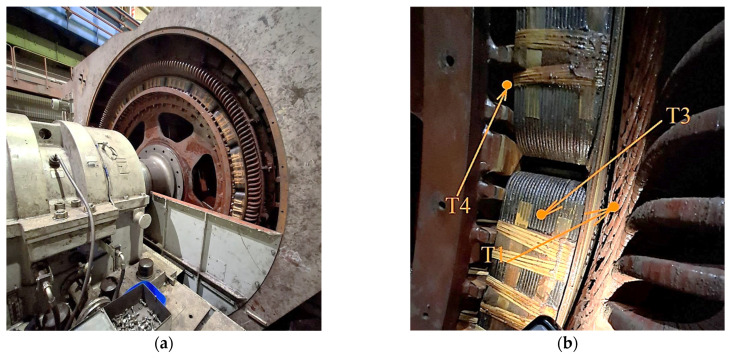
Photograph of motor VEM DMMYZ 3867-20V with a removed casing (**a**) and of windings (**b**) with indication of temperature measurement points.

Figure 12 shows the photographs of the studied motor with removed casing. There are the general view of the stator and rotor (Figure 12a) and the points at which temperature was measured by the pyrometer (Figure 12b). Temperatures of the stator winding, rotor winding and rotor iron core were measured at points T1, T3, and T4. The stator iron core temperature measurement point is not shown in the photograph because it is not seen in it. The pyrometers have been preliminarily tested by the metrology service. Therefore, the information produced by them is reliable. The intrinsic uncertainty of temperature measurements and the impacts of external factors were not considered. We did not consider contact temperature measurement methods using wireless sensor nodes equipped with communication modules to send results to the control centre. This makes up an independent problem that is not related to the development of the temperature monitor based on the thermal model.

Then, the obtained results were validated. For that, the temperatures for the specific time moments shown in Figure 11a, were compared with the results of measurement at control points shown in Figure 12b. The temperature values registered during the pause at the moment *t*_3_ are given in the first line of Table 6. The results of the direct temperature measurements made with the laser pyrometer at the specified points are given in line 2. Point T2, at which stator iron core temperature was measured, is now seen in the photograph, but the temperature measurements were made, and the result is given in the corresponding cell of the table. Line 3 gives the values of relative difference (error) of the temperature calculation results received from the observer. The maximum difference of the restored and measured temperature values is 8.4%. The error can be explained by the following reasons:

- The measurement of values in line 2 and registration of temperatures from oscillograms were performed in the same conditions, but at a different time, thus it is necessary to account for the error explained by natural change of the motor temperature;
- It is difficult to perform measurements by pyrometer with absolute accuracy because the access to control points is complicated. This is because of the complex design of the motor and safety reasons.

Thus, errors can mainly be attributed to the complexity of sensor placement relative to the measurement point. We assume that other error sources, like ambient temperature fluctuations and redundant sensors, do not affect the precision of temperature measurement.

Taking into account the above, we can state that the results of the temperature restoration for the specific motor masses are adequate compared to the results of the physical measurements. The comparison with the allowable temperature values given in Table 2 demonstrated that, during the analysed workpieces rolling within the studied time period, the temperature is significantly lower than the limit values.

In addition, the following conclusions can be drawn from the analysis of the diagrams from Figure 11:

1. Diagram *T*_St_ *Tst*_St_ showing the stator winding temperature combines two components: a relatively slow heating that occurs during the whole time periods (actually, it is registered by diagram *T*_Ir_St_ of the stator iron core temperature) and rapid changes (temperature variation) during the passes. During rolling (for example, within the time period *t*_1_–*t*_2_) the motor is heated, and during the pause (within the time period *t*_2_–*t*_3_) it is cooled.
2. Stator iron core temperature *T*_Ir_St_ and rotor iron core temperature *T*_Ir_R_ at moment *t*_3_ are lower than the temperatures of the respective windings by 1–6 °C and range within 317–321 °K (44–48 °C). The thermal state of the motor is characterised by the stator temperature that is approximately three times lower than the limit value for insulation class F (155 °C in Table 1).
3. The given diagrams indirectly confirm that the temperature depends not only on the load, but on the speed as well [98,99]. However, the impact of speed changes at high loads is low for the installed low-speed motors compared to the impact of the load currents.
4. Maximum range Δ*T*_St_ of stator temperature variation in the diagram in panel 1 is about 1.5 °C. Temperature variations correspond to the diagram of passes (Figure 4a). This confirms the conclusion drawn in [63]: the change of the motor torque variation range has a larger impact on the stator temperature (Figure 11a), but a smaller impact on the rotor temperature (Figure 11b). The variations of current and speed result in the variation of losses in the stator and rotor.

The results are important for the evaluation of motor thermal state because the earlier developed two-mass thermal model could not provide such information. The literature review showed that there have been no studies into the heating of separate parts of motors measured in on-line mode.

### 6.2. Analysis of UMD and LMD Motor Winding Temperatures

**Figure 13:**
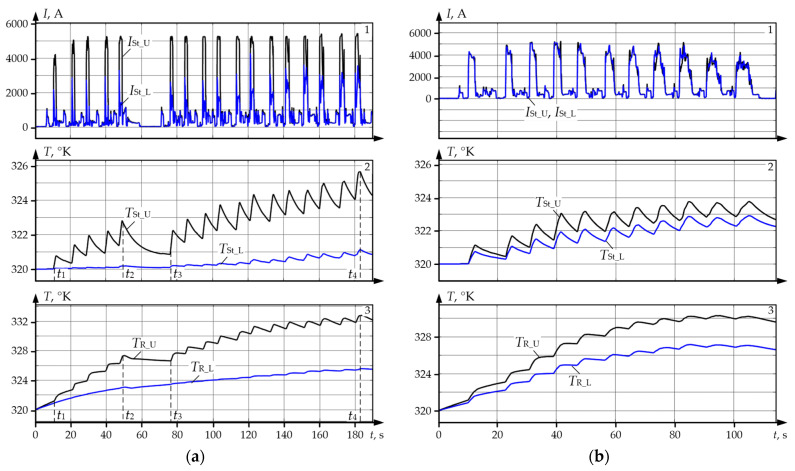
Oscillograms of stator currents *I*_St_U_ and *I*_St_L_ (panel 1), stator winding temperature *T*_St_U_ and *T*_St_L_ (panel 2), and rotors *T*_R_U_ and *T*_R_L_ (panel 3) of UMD and LMD motors during rolling with a 10% ski (**a**) and without ski (**b**).

Figure 13a gives oscillograms of motor winding temperatures during a rolling cycle and the same temperatures restored from the datasets corresponding to the oscillograms in Figure 4a. Black colour shows the diagrams for UMD (symbols with code U), blue colour is for LMD (code L). As is has been mentioned, they were received during rolling with a large pre-set ski; thus, the oscillograms of stator currents *I_St_U_* and *I_St_L_* of UMD and LMD motors in panel 1, Figure 13a differ. Figure 13b gives similar dependences built from the datasets corresponding to Figure 4b, i.e., rolling without ski. For this case, the oscillograms of currents *I*_St_U_ and *I*_St_L_ coincide.

Temperature values at specific time moments shown in Figure 13, a are given in Table 7. The analysis of the oscillograms and table data allowed drawing the following conclusions:

1. Rotor windings during the whole rolling cycle are heated more compared to the stator windings. In both figures during the whole time period, *T*_R_U_ > *T*_St_U_ and *T*_R_L_ > *T*_St_L_. Therefore, the temperature difference is small, around 1–7 °C. This confirms the conclusion drawn in [45]: “temperature correlation between the stator and rotor is relatively constant that highlights the fundamental nature of their heat interaction”.
2. Assumptions on the impact of load currents on motor heating made from the results of the analysis of oscillograms from Figure 4 are confirmed by the given temperature diagrams. With different loading of UMD and LMD motors (Figure 13a), stator winding temperature *T*_St_U_ of UMD motor at the end of the rolling cycle (at time moment *t*_4_) is higher than temperature *T*_St_L_ of LMD motor by approximately 5° (325.7 °K and 321 °K). At equal loads (Figure 13b) stator temperature differ by approximately 0.5 °C. Rotor temperature values differ by 2.3° (326.5 and 328.8 °K).
3. Actual temperature values of the stator and rotor vary within a range of 320–333 °K (47–60 °C) that is close to the ambient temperature. They are two times lower than the limit values given in Table 2. This is quite explicable, as the rolling time is small—about 3 min. Below, the authors show that at a longer time period, the temperature increases considerably, which confirms the importance of its control.

## 7. Summary of the Results

**Figure 14:**
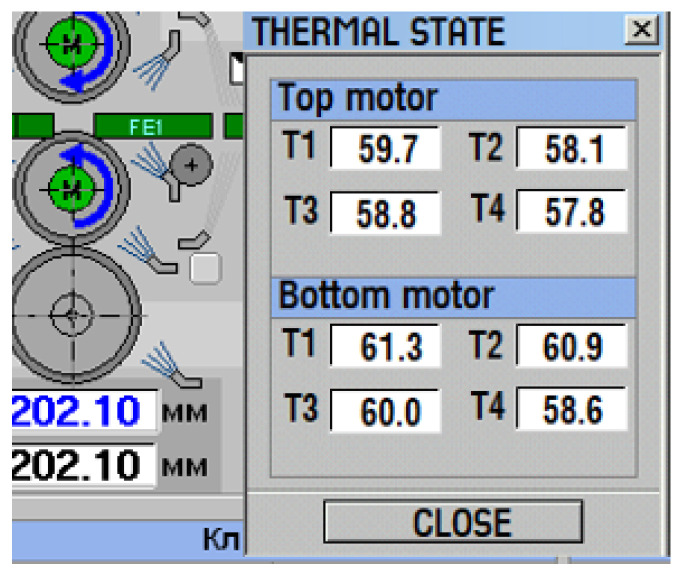
Pop-up window on operator monitor at rolling mill 5000 informing about motor temperature.

The developed temperature observer was introduced into operation at the rolling mill 5000. The information on the thermal state of the motors was visualized. A popup window on the operator screen warns about the unacceptable overheating (Figure 14). This window appears when motor temperatures are approaching the set limit values; in addition, it can pop-up at the operator’s command.

### 7.1. Emergency Prevention

The signal from the observer output is used to warn the personnel and to protect the motor protection from overheating. The authors of [100] mentions that “control of the thermal state windings of the electrical machines is the basis for the protection from unacceptable overheating”. It is well-known that “thermal protection extends the insulation life and prevents it from active ageing and damage from heating loads, restricts the motor overheating from overcurrents” [101]. At the manned facilities, protection consists of two stages: alarm and shutting down. At the unmanned facilities it is recommended to implement protection in one stage envisaging shutting down.

According to the electrical installation code, the electrical motors with forced ventilation require installing the protection that triggers an alarm and/or shuts off the electrical motor when the temperature increases, or the cooling system fails [102]. Electrical motors with water cooling of windings and active stator steel, as well as with in-built air coolers cooled by water must have protection that produces a warning when the water flow falls below the pre-set value and that shuts down the electrical motor when water flow stops [103].

Thus, the signal from the observer allows setting the motor protection in two emergency modes:

- When the system of forced water cooling fails, it is required to shut down the motor after rolling the workpiece;
- When the phase that caused overheating fails but does not require immediate shut-down of the motor, it is allowed to continue operation till the end the whole batch rolling.

Indeed, both cases require on-line control of temperature.

### 7.2. Temperature Analysis During Rolling of Two Batches of Workpieces

**Figure 15:**
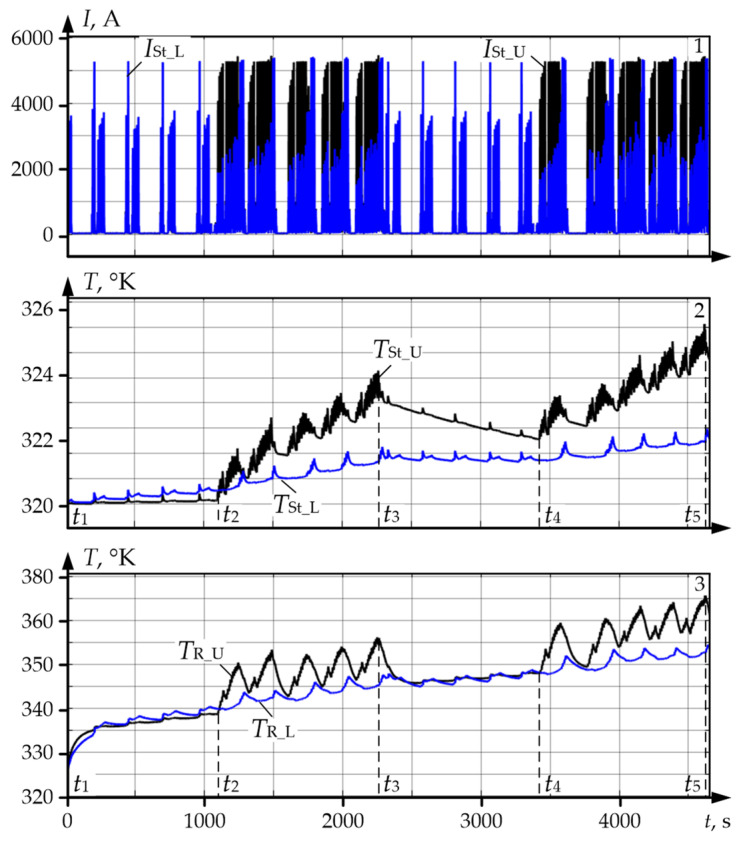
Oscillograms of currents and winding temperatures of UMD and LMD motors during rolling of 10 workpieces; symbols correspond to those shown in Figure 14. Panel 1—stator current *I*_St_U_—black, *I*_St_L_—blue; panel 2—stator winding temperatures *T*_St_U_ and *T*_St_L_; panel 3—rotor winding temperatures *T*_R_U_ and *T*_R_L_.

To summarize the study results, below is the analysis of heating of stators and rotors windings of UMS and LMD motors during a long time period. Figure 15 shows the oscillograms of currents and temperatures of motors during the rolling of two batches consisting of 5 workpieces each. A total of 10 slabs were rolled, and the experiment lasted 1 h 10 min. During time intervals *t*_1_–*t*_2_ and *t*_3_–*t*_4_, the first and the second batches were subsequently rolled in roughing, each in seven passes. Then, during finished product rolling, within time periods *t*_2_–*t*_3_ and *t*_4_–*t*_5_ each workpiece was rolled individually in 19 passes. The rolling was performed with a large ski, thus the UMD motor currents shown in panel 1 in black in each pass considerably exceed the LMD motor currents shown in blue. The stator and rotor winding temperatures registered at the specific time moments are given in Table 8.

The following conclusions are drawn from the shown oscillograms:

1. At the stages of roughing motors are not heated to a great extent. Thus, during the time interval *t*_1_–*t*_2_ stator winding temperatures *T*_St_U_ and *T*_St_L_ (panel 2) are almost constant and are close to 330 °K (57 °C). Rotor winding temperatures *T*_R_U_ and *T*_R_L_ (panel 3) vary within the range from 335 °K to 340 °K. In the similar interval *t*_3_–*t*_4_ temperature *T*_St_L_ of the LMD stator winding is within 340 °K, and temperature *T*_St_U_ of the stator winding even falls from 351 °K to 343 °K. An insignificant change of temperature within these time periods is explained by long pauses caused by cooling-down of workpieces on the roller table, as shown in Figure 1b.
2. During the time intervals *t*_2_–*t*_3_ and *t*_4_–*t*_5_ of the finished product rolling average temperature values of the UMD and LMD stator windings significantly increase. During each pass the heating and the subsequent cooling occur, and the processes are almost exponential. During the time interval *t*_2_–*t*_3_ the average temperature *T*_St_U_ of the UMD motor (panel 2) increased by 21° (from 330 °K to 351 °K), during the time interval *t*_4_–*t*_5_ it increases by 29° from 343 °K to 372 °K.
3. Similar heating processes occur in the rotor windings (panel 3). Rotor temperature values during the whole time period vary within 325–371 °K (52–98 °C) for UMD and 325–355 °K (52–82 °C) for LMD.
4. The shown oscillograms confirm that stator and rotor windings temperatures are lower than the permitted limit values given in Table 1 and Table 2. However, the maximum rotor winding temperature of UMD motor equals 98 °C that is close to the limit value (100 °C, Table 2). Maximum temperature of the stator winding equals 99 °C and also approaches the risk zone (limit value equals 120 °C). This confirms the following conclusion:
  4.1 The constant control of motor temperature and warning about overheating are reasonable.
  4.2 At a specific moment the stator and rotor windings temperatures are almost the same. The stator and rotor iron core temperature values are close, as well. Thus, the requirements to provide a continuous temperature control of the four masses of the motor are reasonable.
  4.3 As the rolled workpieces are hard, the performed analysis allows inferring that it is possible to operate motors without limiting the workpieces dimensions. Therefore, on-line monitoring of all four masses of each motor must be performed.

The application of the developed four-mass heating model is obligatory to control the thermal state of motors with air-to-water cooling. This is related to the fact that if the cooling system fails (liquid pressure reduces or it shuts down), stator and rotor iron core can heat quicker than the stator winding. This confirms the need of on-line controlling of the temperatures of all four masses of the electrical motor that can be performed by the developed observer. The development or improvement of air- and water-cooling systems was outside the scope of this article. These problems are, however, highly relevant and can be addressed in the future.

The developed temperature observer has the following advantages:

- Universal application, because the authors used unified blocks from the Simscape Thermal Models library;
- High immunity to interference, due to the digital transmission of data the observer can be operated even in the conditions of high electromagnetic interference;
- No maintenance is required because it does not wear, it is part of the software.

### 7.3. Results and Introduction Prospects

Due to the introduction of the developed temperature observer at rolling mill 5000 it is possible to:

1. Prepare a report on the temperatures of specific masses of the motor in real time. They can be used for the solution of the following tasks:
  - Active control of the cooling system;
  - Improving thermal protection system;
  - Statistical analysis of trends in the motor thermal state changes.
2. Reduce costs for the maintenance and repairs of motors because of timely failure prevention. Make up optimal preventive maintenance and repair schedules.
3. Expand the types of workpieces and increase the productivity of the mill by increasing the cobbing and speed of rolling under reliable thermal control.

The developed thermal state observer based on the motor four-mass thermal model is recommended for the introduction at different rolling mills. It reduces the overheating risk, prevents emergency shutting down of the motors, and provides for positive technical and economic effect in the following cases:

- When a new rolled sheet or stripe types are introduced;
- When the rolling modes of the workpieces are optimized;
- To prevent the emergencies caused by the deterioration of thermal regimes;
- As part of digital twins and digital shadows to build technical state monitoring electromechanical systems for rolling mills.

It is recommended to use the developed technical solution in industrial electrical drives. The advantages of the observer and of the four-mass model based on the Simscape Thermal Model blocks are its simplicity and no personnel training. Another advantage is the experimental determination of the minimum quantity of the model parameters that distinguishes it from the known models considered in the introduction.

This article only reviews the tests carried out on VEM DMMYZ 3867-20V motors installed on the horizontal stand of the 5000 mill. However, the suggested thermal state monitor can be used for a wide range of synchronous motors with electromagnetic excitation that rely on the four-mass model presented in Figure 7. Since this model is based on the versatile Simscape Thermal Models modules, the monitor can be modified to control the temperature of asynchronous motors. Also note that apart from the developed four-mass model, the monitor can make use of a simpler two-mass thermal model (Figure 5). This is confirmed by the test results of these models in 6000 kW electric drives of the tandem cold rolling mill published in [35]. The usage of more complex models, e.g., the seven-mass thermal model mentioned above [94], is possible. However, these problems require additional studies.

The next stage of the research may involve the development of an overheating protection system for motors based on the developed monitor. It is also feasible to test the monitor on the rolling mill whose motors are fitted with stationary winding temperature sensors. This shall help evaluate the reliability of temperature recovery by the monitor in various operating modes.

The area in question has great development prospects. As mentioned in the introduction, thermal forecasting based on time-series processing methods; in particular, Long Short-Term Memory (LSTM) is one of its key components. This time-series forecasting method uses recurrent neural networks (RNN) with a special cell type that can memorize dependencies over large time intervals. LSTM is a good choice for problems that rely on long-term dependencies, including those used in the monitoring of the thermal state that changes over long periods of time.

To confirm this, we should mention the results published in recent years. Thus, the authors of [104] developed a technical condition evaluation method for airplane gas turbine engines using LSTM and recurrent neural networks (RNN). These models were trained based on the error back-propagation-through-time algorithm to facilitate precise gas turbine engine parameter forecasting. The authors of [105] presented the evaluation of the thermal fault of a marine diesel engine based on the LSTM algorithm, which is a simulation model of a diesel engine generated using the MATLAB modelling methods and the Simulink platform. The authors of [106] developed and tested an LSTM-based algorithm for the assessment of the remaining useful life (RUL) of Li-ion batteries. The data pre-processing stage is followed by feature extraction to select the parameters that detect the deterioration of battery performance. The development of technical condition evaluation and RUL assessment procedures for electrical equipment based on the processing of time-series data was covered in [107,108].

The authors of [109] propose forecasting temperature for thermal tests in the aerospace sector based on the physical and hybrid LSTM models. They present a hybrid approach to modelling that combines the physical model with a network and the LSTM. The LSTM model is trained using historical data to identify complex heat exchange dynamics between modules. This ensures better generalizability of the existing models. The thermal state forecasting of transformers based on ISSA-LSTM is also worth mentioning [110]. The authors suggest a new forecasting model for the temperature of the upper layer of transformer oil that combines the improved sparrow search algorithm (ISSA) and the LSTM. They claim that this model can improve forecasting accuracy and overcome the problems of labour-intensive parameter adjustment.

Thus, forecasting technical condition and service life based on the LSTM is deemed a promising research area. This brief literature overview shows that the approach in question is used to assess and forecast thermal models of various equipment. However, there is no data in the available publications on its usage for the analysis of thermal modes of motors. This area is highly relevant for high-power synchronous motors of rolling mills, and therefore should be developed further.

## 8. Conclusions

1. The importance of the constant control of the motor thermal state is shown by the example of electrical drives of the horizontal stand of plate mill 5000. The performed literature review showed that the development of the on-line temperature monitoring systems does not meet the requirements of the industry. It is difficult to install physical devices at the rotating parts of the motor. Known sensorless measurement systems require the use of models and thermal circuits the parameters of which are difficult to determine during operation. Thus, the development of the temperature observer based on the four-mass heating model using the Simscape Thermal Models library domains that is part of the MATLAB Simulink software package is reasonable.
2. The method for calculating thermal loads has been developed that allows performing an automatic check of the motor heating based on the datasets obtained during the rolling. Generally, the method includes the following:
  - Preparation and storage of the datasets that characterise the motor thermal state and were obtained during the measurements by the IbaPDA (or another) system installed at the mill;
  - Export of the data to the MATLAB file, and testing of the algorithms of temperature recovery for specific masses of the thermal model using the hardware-in-the-loop (HIL) simulation;
  - Calculation of thermal parameters, and validation of the results by comparison for the direct measurement of the temperature of specific masses at the motor.
  The method can be applied to automatically calculate the temperature in any time interval from the data stored in the database or measured on-line.
3. The developed thermal state observer can be used to control the temperatures of the stator and rotor windings and iron cores. A four-mass motor heating model has been developed based on the Simscape Thermal Models library domains. An HIL simulation has been used for setting the observer algorithm and specification of the model parameters. The observer introduction does not require complicated mathematical methods and computational algorithms which is important for the adoption at industrial facilities.
4. By the example of the motor reversible stand of rolling mill 5000 the authors have checked the adequacy of the results of temperature recovery for specific masses. For that they were compared with the results of the measurements at control points performed by lase pyrometer. The error of the restored values does not exceed 8.4% and is satisfactory. The conducted experiment confirms that the model provides reliable results and allows recovering temperature from the data measured right during the rolling process.
  Note: The analysis of heating of specific thermal masses at the rolling mill motors formed during rolling was performed for the first time.
5. The authors have compared the temperatures of motor windings at the upper and lower rolls of the horizontal stand during the rolling cycles with different initial considerations: with the pre-set mismatching of speeds by 10% that is required for the ski formation and with equal speeds (for rolling without ski). The following conclusions have been made:
  - Different loads of the motors in one pass and during the whole rolling cycle result in different temperature of the UMD and LMD motors within several degrees;
  - To provide equal thermal regimes, it is expedient to adjust the speeds and loads of the motors.
6. Through analysis of the winding temperatures during a long time interval of continuous rolling (1 h 10 min), it has been confirmed that the motor thermal mode corresponds to the norms. However, the temperature of the UMD motor rotor winding approaches the limit value (100 °C). Maximum temperature of the stator winding is close to 100 °C, as well, and is within the risk zone.
7. The rolled steel belongs to hard steels, and the limit values for the insulation class are not hit, so the authors recommend rolling without restrictions, but constant temperature control should be maintained. To optimize the thermal regimes for the extended grades of steels, it is recommended to develop a smart load control system based on the torque observers installed at the UMD and LMD shafts.
8. The developed observer is recommended for the introduction at the operating rolling mills and can be applied in industrial electrical drives. It can be used for improving control algorithms for air-to-water cooling systems and for the development of the two-stage protection system for motors. The technical and economic effect will be positive due to reduction of the production risks with minimum introduction costs.

## Figures and Tables

**Table 1:** Limit temperature values for different winding insulation classes.

Thermal Resistance Class	Temperature, °C
Y	90
A	105
E	120
B	130
F	155
H	180

**Table 2:** Limit temperature values for electrical machine elements.

Element	Temperature, °C
Stator winding	120
Stator iron core	110
Rotor winding	100

**Table 3:** Technical characteristics of motor VEM DMMYZ 3867-20V.

Type	Synchronous
Rotor excitation	salient-pole
Poles quantity	20
Manufacturer	VEM Sachsenwerk GmbH
Capacity	12,000 kW
Nominal voltage	3300 V
Nominal speed	70 rpm
Maximum speed	115 rpm
Nominal frequency	10 Hz
Maximum frequency	19.2 Hz
Overload at minimum speed	225% within 30 s
Current at nominal speed and 100% load	2460 A
Maximum current	6000 A
Nominal torque	1910 kNm
Maximum torque during rolling	3820 kNm (200%)
Maximum torque during overload	4298 kNm (225%)
Shut-off torque	5252 kNm (275%)
Power factor	1
Excitation voltage at no load	138.7 V
Nominal excitation voltage	220 V
Excitation current at no load	568 A
Nominal excitation current	898 A
Excitation current at maximum load	1680 A
Insulation class	F
Cooling type	IC86W

**Table 4:** Thermal masses of Simscape Thermal Models package used for model development.

Name	Code	Relations	Note
Thermal mass (stator, rotor windings and stator, rotor iron cores)	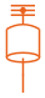	$Q=c\cdot m\frac{dT}{dt}$	*Q* is the heat flow; *c* is the specific heat of the mass material; *m* is the mass; *T* is the temperature; *t* is time
Thermal conductivity description block	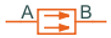	$Q=k_{1}\frac{A}{D}(T_{A}-T_{B})$	Q is heat flow in W; $k_{1}$ is thermal conductivity of the material between masses (insulation), W/(m °K); *A* is the area through which heat is transferred; *D* is the thickness of the layer through which heat is transferred; $T_{A}$ is the temperature of layer A (mass 1), °K; $T_{B}$ is the temperature of layer B (mass 2), °K
Convection description block	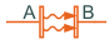	$Q=k_{2}A(T_{A}-T_{B})$	$k_{2}$ is heat transfer by convection coefficient, W/(K∙m^2^).
Block for setting the externally controlled heat connected to the mass	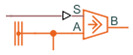		For example, heating from thermal effect of the current in winding or energy formed in the iron core
Thermal resistance	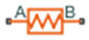	R·Q=∆T $R=\frac{D}{k\cdot A}=\frac{1}{h\cdot A}=\frac{1}{r\cdot A\left(T_{A}^{2}+T_{B}^{2}\right)\left(T_{A}+T_{B}\right)}$	*R* is the thermal resistance; *Q* is the heat flow rate; Δ*T* is the temperature difference between layers; *D* is material thickness, that is, distance between layers; *A* is area normal to the heat flow direction; *k* is thermal conductivity of the material; *h* is convection heat transfer coefficient, *r* is radiation coefficient; *T*_A_ and *T*_B_ are temperatures at ports A and B, respectively
Thermal Sensors (mass temperature sensors block)	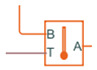	T—Temperature measurement, K physical signal; A—Sensor inlet thermal; B—Sensor outlet thermal	Т—Physical signal output port for temperature measurement; А—Thermal conserving port; B—Thermal conserving port; Measured temperature $T=T_{A}-T_{B}$ $Q=k_{2}A(T_{A}-T_{B})$

**Table 5:** Parameters of thermal model.

Parameter	Code	Value
Stator iron core weight	$m_{2}$	70,000 kg
Rotor iron core weight	$m_{4}$	90,000 kg
Stator winding weight	$m_{1}$	5000 kg
Rotor winding weight	$m_{3}$	2000 kg
Stator winding heat absorption	$C_{1}$	385 J/(kg °K)
Rotor winding heat absorption	$C_{3}$	385 J/(kg °K)
Stator iron core heat absorption	$C_{2}$	447 J/(kg °K)
Rotor iron core heat absorption	$C_{4}$	447 J/(kg °K)
Contact area of the stator winding and iron core	$A_{11}$	3 m^2^
Contact area of the rotor winding and iron core	$A_{12}$	1 m^2^
Heat transfer coefficient for the stator winding—stator iron core	$K_{11}$	200 W/(m °K)
Heat transfer coefficient for the rotor winding—rotor iron core	$K_{12}$	600 W/(m °K)
Stator cooling area	$A_{21}$	10 m^2^
Rotor cooling area	$A_{22}$	4 m^2^
Heat removal coefficient for stator	$K_{21}$	1500 W/(m °K)
Heat removal coefficient for rotor	$K_{22}$	1500 W/(m °K)
Electrical resistance of stator winding	$R_{0}$ stator	0.07 Ohm
Electrical resistance of rotor winding	$R_{0}$ rotor	0.3 Ohm
Thermal resistivity constant for windings	α	0.0043 1/°K

**Table 6:** Temperature values from control points registered by the observer at *t*_3_ time moment and measured by pyrometer, °C.

Measurement Tool	Measurement Point			
	Stator Winding (T1)	Stator Iron Core (T2)	Rotor Winding (T3)	Rotor Iron Core (T4)
Observer	47.5	47.5	53.5	47.1
Pyrometer	45.0	44.5	49.0	45.5
Error, %	5.2	6.3	8.4	3.6

**Table 7:** Stator and rotor temperature at specific time moments for diagrams from Figure 13a.

Time	Temperature							
	UMD Motor				LMD Motor			
	*T*_St_U_		*T*_R_U_		*T*_St_L_		*T*_R_L_	
Temperature unit	°K	°C	°K	°C	°K	°C	°K	°C
*t*_1_	320	47	321	48	320	47	321	48
*t*_2_	322.8	49.8	321.2	54.2	320	47	323.1	50.1
*t*_3_	320.9	47.9	326.8	53.8	320	47	323.6	50.6
*t*_4_	325.7	52.7	332.8	59.8	321	48	325.5	52.5

**Table 8:** Temperature values at specific time moments for UMD and LMD motors.

Time	Temperature, °C							
	Stator Windings				Rotor Windings			
	*T*_St_U_		*T*_R_L_		*T*_R_U_		*T*_R_L_	
Temperature unit	°K	°C	°K	°C	°K	°C	°K	°C
*t*_1_	330	57	330	57	325	52	325	52
*t*_2_	330	57	333	60	338	65	340	67
*t*_3_	351	87	339	66	356	83	345	72
*t*_4_	343	70	339	66	348	75	348	75
*t*_5_	372	99	340	67	371	98	355	82

## Data Availability

The original contributions presented in this study are included in the article. Further inquiries can be directed to the corresponding author.
